# The Effects of Broiler Breeder Dietary Vitamin E and Egg Storage Time on the Quality of Eggs and Newly Hatched Chicks

**DOI:** 10.3390/ani10081409

**Published:** 2020-08-13

**Authors:** Jun Yang, Xuemei Ding, Shiping Bai, Jianping Wang, Qiufeng Zeng, Huanwei Peng, Yue Xuan, Zuowei Su, Keying Zhang

**Affiliations:** Institute of Animal Nutrition, Key Laboratory for Animal Disease-Resistance Nutrition of China Ministry of Education, Sichuan Agricultural University, 211 Huimin Road, Wenjiang District, Chengdu 611130, China; yangjun0612@163.com (J.Y.); shipingbai@sicau.edu.cn (S.B.); wangjianping@sicau.edu.cn (J.W.); zqf@sicau.edu.cn (Q.Z.); phw@sicau.edu.cn (H.P.); 71128@sicau.edu.cn (Y.X.); wzs698@126.com (Z.S.)

**Keywords:** breeder dietary vitamin E, egg storage, hatchability, antioxidant status

## Abstract

**Simple Summary:**

Broiler breeder dietary vitamin E supplementation has been indicated to enhance the antioxidant status of egg yolks, embryos, and newly hatched chicks. However, knowledge of the relationships involving breeder dietary vitamin E, the egg storage time, and the quality of the eggs and newly hatched chicks in poultry, especially in broiler breeders, is still limited. Here, we aimed to provide important evidence regarding broiler breeder dietary vitamin E and egg storage time intervention in egg characteristics and hatchability and the antioxidant status of the egg yolks and newly hatched chicks. Our results showed that prolonged egg storage time (14 vs. 0 d) increased the embryonic mortality, decreased the hatchability, and impaired the antioxidant status of egg yolks and newly hatched chicks, while increasing the broiler breeder dietary vitamin E levels (100 vs. 6 mg/kg) improved the livability of embryos and the antioxidant status of newly hatched chicks in the case of long-term egg storage. These findings suggested that broiler breeder dietary vitamin E could be applied in extending the storage time of breeder eggs.

**Abstract:**

This study was conducted to investigate the effects of broiler breeder dietary vitamin E and egg storage time on the egg characteristics, hatchability, and antioxidant status of the egg yolks and newly hatched chicks. A total of 512 71-week-old Ross 308 breeder hens were fed the same basic diets containing 6 or 100 mg/kg vitamin E for 12 weeks. During this time, a total of 1532, 1464, and 1316 eggs were independently collected at weeks 8, 10, and 12, respectively, and subsequently stored for 0 or 14 d before hatching. The outcomes from three trials showed that prolonged egg storage time (14 vs. 0 d) negatively affected (*p* < 0.05) the egg characteristics, hatchability traits, and the yolk total antioxidant capacity (T-AOC) (*p* < 0.05). Chicks derived from the stored eggs exhibited higher malonaldehyde (MDA) and T-AOC in the serum and yolk sac (*p* < 0.05). Broiler breeder dietary vitamin E (100 vs. 6 mg/kg) increased (*p* < 0.05) the hatchability and the antioxidant status of the yolks as indicated by a higher α-tocopherol content and T-AOC and lower MDA level (*p* < 0.05). The supplementation of vitamin E also remarkably increased (*p* < 0.05) the total superoxide dismutase (T-SOD) activity (yolk sac, weeks 8 and 12) and T-AOC (serum, weeks 8, 10, and 12; yolk sac, weeks 8 and 12) and decreased (*p* < 0.05) the MDA content of chicks (yolk sac, week 10; serum, week 12). Interactions (*p* < 0.05) were found between the broiler breeder dietary vitamin E and egg storage time on the hatchability and antioxidant status of chick tissues. Broiler breeder dietary vitamin E (100 vs. 6 mg/kg) increased (*p* < 0.05) the hatchability and the T-AOC in the serum and liver of chicks, and decreased (*p* < 0.05) the early embryonic mortality and the MDA content in the yolk sacs of chicks derived from eggs stored for 14 d but not for 0 d. In conclusion, prolonged egg storage time (14 vs. 0 d) increased the embryonic mortality, decreased the hatchability, and impaired the antioxidant status of egg yolks and newly hatched chicks, while the addition of broiler breeder dietary vitamin E (100 vs. 6 mg/kg) could partly relieve these adverse impacts induced by long-term egg storage.

## 1. Introduction

The hatchability and quality of chicks are the most important indices for the fertility evaluation of broiler breeders. Fertilized egg storage is a frequent practice in commercial poultry production. The egg storage duration mainly depends on the hatchery capacity and the variable market demand of newly hatched chicks. Generally, the hatchability is not problematic when eggs are stored for less than 7 days [1,2,3,4]. However, egg storage times beyond 7 days is shown to increase the mortality of embryos, and decrease the hatchability [1,2,3,5,6] and quality of newly hatched chicks [6,7]. A previous study indicated that prolonged egg storage time (21 vs. 4 d) significantly up- or down-regulated the relative expression of the pro-apoptotic gene CASP9 (Caspase 9, apoptosis-related cysteine peptidase) and oxidative stress gene SOD2 (superoxide dismutase 2, mitochondrial) in blastodermal cells [8]. Ebeid et al. [9] reported that prolonged egg storage time from 4 d to 14 d decreased the hatchability of set/fertile eggs, increased the embryonic mortality, and negatively impacted the antioxidant status of newly hatched chicks. Oxidative stress appears to be a potential factor that caused lower hatchability and higher mortality in long-term egg storage. In this case, the supplementation of breeder dietary antioxidants may be effective to minimize embryonic mortality induced by long-term egg storage.

In broiler breeders, vitamin E is a necessary micronutrient, and the recommended level is 6 mg/kg in the diet [10]. Previous studies demonstrated that broiler breeder dietary vitamin E supplementation (0–500 mg/kg) did not affect the breeder performance and egg characteristics [11,12,13], however, did improve the antioxidant status of breeders due to the antioxidative characteristics [14,15]. The breeder dietary vitamin E could be transported to the egg yolk [16,17,18], which was beneficial for the egg yolk and embryo to resist against lipid peroxidation. During the embryonic development, especially from d 12 of incubation to d 1 after hatching, the yolk vitamin E concentration gradually decreased, while the brain vitamin E was maintained at a relatively low level.The content of vitamin E in the liver, yolk sac membrane, and adipose tissue increased after d 15 of incubation [19]. Surai et al. [20] indicated that broiler breeder dietary vitamin E supplementation (365 vs. 147 mg/kg) increased the content of vitamin E in the yolk sac membrane, liver, brain, and lungs ofa 16-d-old embryo and 1-d-old chicks, which ultimately decreased the susceptibility to lipid peroxidation. Tsai et al. [21] reported that increasing levels of broiler breeder dietary vitamin E, i.e., 0, 40, 80, 120, and 160 mg/kg, could increase the fertility and hatchability of set eggs, the brain vitamin E content, and the superoxide dismutase (SOD) activity of chicks, and could decrease the brain MDA and reactive oxygen species (ROS) levels of chicks. However, other authors did not find that the broiler breeder dietary vitamin E level (0–150 mg/kg) had any effect on the fertility and hatchability [11,12,13,16,18]. Modern broiler breeder production usually faces a variety of stressful environments, in this case, whether or not the recommendation of broiler breeder dietary vitamin E by NRC (1994) is low, and higher dietary vitamin E could maintain stable reproductive performance.

Interestingly, no work has been done to evaluate the effects of broiler breeder dietary vitamin E and egg storage time on the hatchability and antioxidant status of newly hatched chicks. Therefore, our hypothesis for this study was that the addition of vitamin E in the broiler breeder diet could improve the quality of eggs and newly hatched chicks after long-term storage by improving the antioxidant status of both the eggs and newly hatched chicks. Accordingly, the objective of this study was to evaluate the effects of broiler breeder dietary vitamin E and egg storage time on the quality of eggs and newly hatched chicks.

## 2. Materials and Methods

All animal procedures associated with this study were approved by the Animal Care and Use committee, Sichuan Agricultural University (Ethic Approval Code: SICAUAC201805-5; Cheng, China).

### 2.1. Experimental Design and Diets

A total of 512 71-week-old Ross 308 breeder hens were assigned to two dietary treatments with eight replicates of 32 hens. Breeder hens were fed a basal mash diet (Table 1) with the addition of vitamin E at 6 or 100 mg/kg (the determined values were 10.5 and 106.2 mg/kg, respectively) for 12 weeks. The breeders were bred following the Ross 308 broiler breeder management guidelines. During the trial, the average daily feed intake was 140 g/day per hen and 120 g/day per rooster. A photoperiod of 16L:8D was used, and water was provided ad libitum.

The egg quality and hatchability evaluation were conducted using a 2 × 2 factorial arrangement with two broiler breeder dietary vitamin E levels (6 or 100 mg/kg) and two egg storage times (stored 0 or 14 d) with six replicates per treatment at weeks 8, 10, and 12. At each week, the eggs per hen treatment were collected over a 5-d period and then allocated into two groups: one group containing half of the collected eggs that were immediately incubated at the last day of egg collection (stored 0 d), and the other group containing the remaining half of the eggs, which were stored for 14 days (stored 14 d, turned every 4 h during the storage period until incubation). A total of 1532, 1464, and 1316 eggs were collected at weeks 8, 10, and 12, respectively. The storage temperatures for eggs stored 14 d were different, with22–24 °C for 1–14 d at week 8, 22–24 °C for 1–7 d and 16–18 °C for 8–14 d at week 10, and 16–18 °C for 1–14 d at week 12.

All eggs were incubated in a commercial incubator (Pearl-22, I.P Co., Ltd., Japan). The temperature ofthe dry-bulb was initially controlled at 37.9°Cand gradually decreased down to 36.5 °C as the incubation time increased; the wet-bulb temperature was maintained at 28.2 °C After 19 days of incubation, the eggs were transferred into a hatcher with the dry-bulb set at 36.9°Cand the wet-bulb set at 29.0 °Cuntil incubation.

### 2.2. Egg Characteristics

A total of 24 eggs per treatment stored for 0 d or 14 d (before incubation) were used to measure the egg weight, albumen height, Haugh units, yolk color value, and yolk ratio according to the method of our laboratory [22]. The egg weight, egg yolk color value, Haugh units and albumen height were evaluated using an egg multi tester (EMT-7300, Co., Ltd., Tokyo, Japan). Egg yolk was then separated from the albumen and weighed, and yolk ratio was calculated according to the formula of yolk weight/egg weight.

### 2.3. Egg Weight Loss, Hatchability, and Embryonic Mortality

The weight of all the eggs for incubation was determined before and after the storage for 0 d or 14 d. During the egg incubation, all the eggs were candled at 7, 14, and 19 days of incubation, while the unhatched eggs after 21.5 d of incubation were open to determine the number of infertile eggs and dead embryos, which were divided into three categories according to the stage of embryonic mortality from d 1 to 7 (early), d 8 to 14 (mid), and d 15 to 21.5 (late). The calculations of the egg weight loss during storage, the fertility, the hatchability of set/fertile eggs, the embryonic mortality, the chick weight, and the ratio of healthy chicks were according to the description of Ebeid et al. [9].

### 2.4. Antioxidant Status of Egg Yolk

After evaluation of the egg characteristics, the egg yolk was separated.Four yolks per replicate were mixed and then stored at ‒20°Cfor antioxidant status analyses. Briefly, the yolk samples (0.4 g) were homogenized in 3.6 mL of ethanol for the determination of malonaldehyde (MDA) and in 1.6 mL of physiological saline for the determination of the total superoxide dismutase (T-SOD) and total antioxidant capacity (T-AOC). The homogenates were then centrifuged at 1500× *g* for 10 min at 4 °C to obtain the supernatant fluid. The specific assay kits were purchased from the Nanjing Jiancheng Bioengineering Institute of China. The yolk α-tocopherol concentration was determined using high-performance liquid chromatography [23].

### 2.5. Antioxidant Status of Newly Hatched Chicks

On the day of hatching, one chick per replicate was randomly selected for blood sampling via the jugular vein. The blood samples were centrifuged at 1200× *g* for 10 min at 4 °C to obtain serum samples, which were immediately stored at −20 °C until further analyses. The chicks were then killed by cervical dislocation, and the liver and yolk sac were dissected and stored at −20 °C. The MDA, T-SOD, and T-AOC determination of the yolk sac samples were performed based on the methods for egg yolk. The analyses of MDA, T-SOD, and T-AOC in the serum and liver were performed according to the protocol of specific assay kits (Nanjing Jiancheng Bioengineering Institute, Jiangsu, China).

### 2.6. Statistical Analyses

Data were analyzed by ANOVA as a 2 × 2 factorial using GLM procedures of SPSS 21.0 (SPSS Inc., Chicago, IL, USA). The main effects (broiler breeder dietary vitamin E and egg storage time) and interactions between the two factors were investigated. When the interactions showed significance, Duncan’s significant-difference test was applied. The data are shown as the means and pooled SEM. The results were considered significantly different at *p* < 0.05.

## 3. Results

### 3.1. Egg Characteristics

The results shown in Table 2 indicate that prolonged egg storage time (14 vs. 0 d) decreased the egg weight, albumen height and Haugh units (*p* < 0.05), and increased the yolk color and yolk ratio (*p* < 0.05). No effects of broiler breeder dietary vitamin E or interactions between breeder dietaryvitamin E and the egg storage time were found on the egg characteristics (*p* > 0.05).

### 3.2. Egg Weight Loss, Hatchability and Embryonic Mortality

The data given in Table 3 indicates that prolonged egg storage time (14 vs. 0 d) increased the egg weight loss during storage, in particular for eggs at weeks 8 and 10, which were higher than eggs from week 12 (*p* < 0.05). Eggs stored for 14 d resulted in decreased fertility (except week 12), hatchability of set/fertile eggs, chick weight, healthy chick ratio (except week 12), and healthy chick number per hen (*p* < 0.05), and increased embryonic mortality in the early and late (except week 12) incubation of eggs at weeks 8, 10, and 12 (*p* < 0.05). Broiler breeder dietary vitamin E (100 vs. 6 mg/kg) increased the hatchability of set/fertile eggs, the healthy chick number per hen at week 10 (*p* < 0.05), and the hatchability of fertile eggs at week 12 (*p* < 0.05), and decreased the embryonic mortality in early incubation at week 12 (*p* < 0.05). Interactions between the broiler breeder dietary vitamin E and egg storage time on the hatchability of set/fertile eggs at week 8, the hatchability of fertile eggs at weeks 10 and 12, and the early embryonic mortality at weeks 8 and 12 were significant (*p* < 0.05). No difference (*p* > 0.05) between the broiler breeder dietaryvitamin E levels was observed on the hatchability of set/fertile eggs at week 8, the hatchability of fertile eggs at weeks 10 and 12, and the early embryonic mortality at weeks 8 and 12 when eggs were unstored. However, for eggs stored for 14 d, broiler breeder dietary vitamin E (100 vs. 6 mg/kg) increased the hatchability of set/fertile eggs at week 8, the hatchability of fertile eggs at weeks 10 and 12, and the early embryonic mortality at weeks 8 and 12. The healthy chick numbers per hen were increased due to higher broiler breeder dietary vitamin E (100 mg/kg) for the eggs stored for 14 d, by 3.0, 4.9,and 2.1 chicks in eggs from weeks 8, 10, and 12, respectively.

### 3.3. Antioxidant Status of Egg Yolk

As shown in Table 4, prolonged egg storage time (14 vs. 0 d) decreased the yolk T-AOC at weeks 8, 10, and 12 (*p* < 0.05), and the T-SOD activity of the yolk at week 10 (*p* < 0.05). The broiler breeder dietary vitamin E (100 vs. 6 mg/kg) increased the yolk α-tocopherol content and T-AOC (*p* < 0.05), and decreased the yolk MDA content at weeks 8, 10, and 12 (*p* < 0.05).

### 3.4. Antioxidant Status of Newly Hatched Chicks

The results of the antioxidant status of newly hatched chicks are shown in Table 5. At week 8, prolonged egg storage time (14 vs. 0 d) increased the MDA content and T-AOC in the serum and yolk sac of chicks (*p* < 0.05). Broiler breeder dietary vitamin E (100 vs. 6 mg/kg) increased the T-SOD activity in the yolk sacs and the T-AOC in the serum and yolk sac of chicks (*p* < 0.05). Interactions were found between broiler breeder dietary vitamin E and egg storage time on the T-AOC in the serum and liver of chicks (*p* < 0.05). Broiler breeder dietary vitamin E (100 vs. 6 mg/kg) did not affect the T-AOC in the serum and liver (*p* < 0.05) when eggs were unstored, and increased the T-AOC in the serum and liver of chicks (*p* < 0.05) from eggs, which were stored for 14 d.

At week 10, prolonged egg storage time (14 vs. 0 d) increased the MDA content and T-AOC in the serum of chicks (*p* < 0.05). Broiler breeder dietary vitamin E (100 vs. 6 mg/kg) increased the T-AOC in the serum of chicks (*p* < 0.05) and decreased the MDA content in the yolk sacs of chicks (*p* < 0.05). No interactions (*p* < 0.05) were found on the antioxidant status of newly hatched chicks.

At week 12, prolonged egg storage time (14 vs. 0 d) increased the MDA content in the yolk sacs and the T-AOC in the serum of chicks (*p* < 0.05). Broiler breeder dietary vitamin E (100 vs. 6 mg/kg) increased the T-SOD activity in the yolk sacs and the T-AOC in the serum and yolk sac of chicks (*p* < 0.05), and decreased the MDA content in the serum of chicks (*p* < 0.05). An interaction (*p* < 0.05) was found between broiler breeder dietary vitamin E and egg storage time on the MDA content in the yolk sacs of newly hatched chicks. No difference (*p* > 0.05) between the broiler breeder dietary vitamin E levels was observed on the MDA content in the yolk sacs of chicks derived from unstored eggs. However, broiler breeder dietary vitamin E (100 vs. 6 mg/kg) decreased the MDA content in the yolk sacs of chicks (*p* < 0.05) derived from eggs, which were stored for 14 d.

## 4. Discussion

In the current study, prolonged egg storage time (14 vs. 0 d) increased the egg weight loss during storage and the early embryonic mortality, and decreased the fertility and hatchability of set/fertile eggs, chick weight, and the ratio of healthy chicks, which was consistent with the results of previous studies [1,2,3,5,6,9,24]. Fasenko et al. [25] concluded that the increased embryonic mortality caused by long-term egg storage might be related to the increased egg weight loss during storage. During egg storage, the embryo was surrounded by the microenvironment involved in egg characteristics, which would adversely affect the viability of blastodermal cells or embryos [26]. In agreement with previous studies [27,28], we also found that prolonged egg storage time (14 vs. 0 d) significantly affected the egg characteristics.On the other hand, Mohitiasli et al. [29], Nimalaratne et al. [30], and Darmawan et al. [31] did not observe that prolonged egg storage time had any effect on the egg yolk MDA content as eggs were stored at 4 °C, but increased the yolk MDA content as eggs were stored at 23–27 °C or 29–31 °C. Due to the effect of storage temperature, we found no change of the yolk MDA content between different storage times as eggs were stored at or below 22–24 °C. However, we found that prolonged egg storage time reduced the yolk T-AOC, and prolonged egg storage decreased the concentration of antioxidant substances in the yolks. Therefore, prolonged egg storage time enhanced the potential risk of lipid peroxidation, and this can be detrimental to blastodermal cells as they are closely bound to the egg yolk. Research has suggested that an optimum number of viable embryonic cells was likely required for the initiation of normal embryonic growth and development [32]. Prolonged egg storage could influence the expression of blastodermal apoptotic genes and increase the percentage of apoptotic and necrotic cells [3,5,8,33,34], which leads to increased embryonic mortality and subsequently a decreased ratio of healthy chicks and the hatchability of set/fertile eggs as found in the present study.

The results in our study indicated that the chick weight was decreased by long-term egg storage, which may be due to the reduced embryonic metabolism and growth, and organ development during incubation [4]. Previous studies showed inconsistent effects of long-term egg storage on fertility [6,9,24,35]. Reijrink et al. [36] indicated that, as egg storage time was prolonged, decreased fertility was observed in eggs stored at 18–20 ℃, but not in eggs stored at 16–18 ℃. Similarly, we found that the fertility was decreased for eggs stored for 14 d at weeks 8 and 10, but not at week 12, in which perhaps we classified some dead embryos during storage as infertile eggs at weeks 8 and 10.

Many reports have demonstrated the effects of dietary vitamin E on improving yolk α-tocopherol and reducing yolk MDA [11,29,37,38]. In agreement with these results, we observed that broiler breeder dietary vitamin E improved the yolk α-tocopherol and T-AOC, and decreased the yolk MDA content in our study. The enhanced antioxidant status of egg yolk by broiler breeder dietary vitamin E was shown to greatly protect embryonic development from lipid peroxidation [17]. The recommended levels of broiler breeder dietary vitamin E are 6 or 100 mg/kg by NRC (1994) and parental nutrient requirement of both Cobb and Ross, respectively. For unstored eggs, broiler breeder dietary vitamin E (100 vs. 6 mg/kg) showed no influence on the embryonic mortality and hatchability of set/fertile eggs. However, when the eggs were stored for 14 d, broiler breeder dietary vitamin E (100 vs. 6 mg/kg) decreased the embryonic mortality and increased the hatchability of set/fertile eggs. It suggested that compared with NRC (1994), the current industry recommended dietary vitamin E level (Cobb and Ross) presented a stronger protective effect against environmental stress. Shirpoor et al. [39] and Yonguc et al. [40] reported that oxidative stress significantly increased the protein carbonyl, lipid hydroperoxide, DNA oxidative rate, and cell apoptosis, and in this case, vitamin E could alleviate these adverse effects of oxidative damages. Thus, these results were similar, and we hypothesize that oxidative stress was possibly involved in the influence of long-term egg storage on the embryonic mortality and hatchability and that broiler breeder dietary vitamin E plays a positive role in this process.

We found the results of the fertility, hatchability, embryonic mortality and the ratio of healthy chick were not consistent between weeks 8, 10, and 12, which may be due to the different storage temperatures set at weeks 8, 10, and 12. Previous studies demonstrated that under the same prolonged periods, the necrotic index of the blastoderms and the reduction of hatchability was increased by the increased egg storage temperature [41,42]. Pokhrel et al. [3] observed that the hatchability of eggs stored for 14, 21, and 28 d at 18℃ and 12 °C were 75%, 55%, 17%,and 88%, 78%, 71%, respectively. Similarly, in the present study we found identical trends between the storage temperature and the percentage of embryonic mortality induced by long-term egg storage, with week 8 > week 10 > week 12. This was a possible reason why we observed the decreased fertility and ratio of healthy chicks, and the increased late embryonic mortality at weeks 8 and 10, except for week 12, even when the egg storage time was prolonged to 14 d. Whether vitamin E could relieve the adverse influence of long-term egg storage appears to be related to the impacted extent of storage temperature on embryonic mortality.

The T-AOC reflects the total levels of non-enzymatic antioxidants, such as the carotenoids, vitamin E, selenium, and ascorbic acid [43]. As a stable product of lipid peroxidation, the concentration of MDA indirectly reflects the attacking degree of the free radical. In this study, we observed that prolonged egg storage time (14 vs. 0 d) increased the MDA content and T-AOC in the serum and yolk sac of newly hatched chicks. We speculated that long-term egg storage might damage the antioxidant system, and negatively impact the effective utilization of antioxidants to protect the chick tissues against lipid peroxidation. To the best of our knowledge, little work has been done to evaluate the effect of prolonged egg storage time on the antioxidant status of newly hatched chicks. Only Ebeid et al. [9] reported that the prolonged egg storage at 12 °C could increase the serum MDA content and decrease the serum T-AOC of 1-d-old chicks. These variations in results may be due to the different egg storage temperatures.

Our results demonstrated that increasing the supplementation of broiler breeder dietary vitamin E could enhance the concentration of egg yolk vitamin E (α-tocopherol). Parolini et al. [44] reported that the in ovo injection of vitamin E increased the plasma T-AOC of hatchlings. Surai et al. [20] and Surai [45] indicated that increasing the broiler breeder dietary vitamin E levels improved the vitamin E concentration and reduced the MDA content in chick tissues. In the present study, broiler breeder dietary vitamin E (100 vs. 6 mg/kg) improved the T-AOC and decreased the MDA content in tissues of newly hatched chicks. This suggested that the improved T-AOC possibly benefited from the enhanced non-enzymatic antioxidant (vitamin E) in chick tissues caused by increased breeder dietary vitamin E. As a result, the process of lipid peroxidation was broken, and the MDA level decreased in chick tissues. Similar to the result of the increased T-SOD activity from broiler breeder dietary vitamin E in the current study, the activity of brain T-SOD in 1-d-old chicks increased as the broiler breeder dietary vitamin E increased [21,46]. Under the conditions of hot stress or oxidative stress, dietary vitamin E was more pronounced in alleviating the damage of the antioxidant status of chicks [47,48,49]. In the present study, the observation of the interaction between broiler breeder dietary vitamin E and egg storage time on the MDA content and T-AOC indicated that broiler breeder dietary vitamin E greatly improved the antioxidant status of chicks in the case of long-term egg storage.

## 5. Conclusions

The present study demonstrated that prolonged egg storage time (14 vs. 0 d) increased the embryonic mortality, decreased the hatchability traits, and impaired the antioxidant status of egg yolks and newly hatched chicks, while increasing broiler breeder dietary vitamin E supplementation from 6 mg/kg (NRC, 1994) to 100 mg/kg (Cobb and Ross) could partly relieve these adverse influences induced by long-term egg storage. These findings suggested that broiler breeder dietary vitamin E supplementation could improve the resistance of fertile eggs to long-term egg storage and could be applied in extending the storage time of breeder eggs.

## Figures and Tables

**Table 1:** The composition and nutrient levels of the basal diet (%, as fed-basis).

Items	Amount (%)
Ingredient	
Corn	69.50
Soybean meal, CP = 43%	19.00
Soybean oil	1.00
Limestone	8.25
Dicalcium phosphate, 2H2O	1.14
L-Lysine hydrochloride, Lys.HCl = 98.5%	0.08
Threonine, Thr = 98.5%	0.02
DL-Methionine, Met = 99%	0.11
Sodium chloride	0.30
Mineral and vitamin premix ^1^	0.50
Choline chloride, 50%	0.10
Total	100.00
Nutritional composition	
ME (kcal/kg)	2780.00
CP	13.80
Ca	3.40
Non-phytate phosphorus	0.30
Digestible lysine	0.66
Digestible methionine	0.32
Digestible methionine + cystine	0.53
Digestible threonine	0.46

^1^ Supplied per kilogram of diet: Cu, 20 mg; Fe, 80 mg; Mn, 82.5 mg; Zn, 100 mg; Se, 0.30 mg; I, 1.20 mg; VA, 12,000 IU; VE, 6 or 100 mg; VD3, 4000 IU; VK3, 4.0 mg; VB1, 3.0 mg; VB2, 11.5 mg; VB6, 7.2 mg; VB12, 0.02mg; folic acid, 10.8 mg; pantothenic acid, 21.6 mg; niacin, 47.1 mg; and biotin, 0.6 mg.

**Table 2:** Theeffects of broiler breeder dietary vitamin E and egg storage time on the egg characteristics.

Items	Egg Stored 0 d		Egg Stored 14 d ^1^		SEM	*p*-Value		
	Vitamin E 6 mg/kg	Vitamin E 100 mg/kg	Vitamin E 6 mg/kg	Vitamin E 100 mg/kg		Egg Storage	Vitamin E	Interaction
**Week 8**								
Egg weight, g	65.90	65.92	65.08	65.36	0.247	<0.001	0.762	0.798
Albumen height, mm	6.60	6.42	4.65	4.48	0.141	<0.001	0.531	0.986
Haugh units	78.63	76.81	62.80	59.53	1.206	<0.001	0.304	0.767
Yolk color	6.42	6.39	7.00	7.34	0.130	0.008	0.554	0.481
Yolk ratio, %	32.24	32.60	34.38	33.46	0.187	0.001	0.464	0.102
**Week** **10**								
Egg weight, g	66.55	66.62	65.10	65.16	0.171	<0.001	0.850	0.994
Albumen height, mm	6.55	6.37	4.71	4.21	0.163	<0.001	0.302	0.629
Haugh units	77.54	76.16	60.15	54.63	1.335	<0.001	0.212	0.449
Yolk color	6.43	6.55	7.26	7.01	0.059	<0.001	0.591	0.136
Yolk ratio, %	32.42	32.84	33.77	34.10	0.207	0.005	0.368	0.913
**Week** **12**								
Egg weight, g	66.29	66.40	65.35	64.84	0.135	<0.001	0.468	0.260
Albumen height, mm	6.47	6.37	4.68	4.82	0.161	<0.001	0.951	0.706
Haugh units	78.97	77.83	62.23	62.45	1.169	<0.001	0.846	0.775
Yolk color	6.20	6.31	7.49	6.89	0.121	0.001	0.321	0.159
Yolk ratio, %	32.60	32.49	33.47	33.72	0.192	0.013	0.857	0.641

^1^ The temperature for eggs stored 14 d was controlled at 22–24 °C for 1–14 d at week 8, 22–24 °C for 1–7 d, and 16–18 °C for 8–14 d at week 10, and 16–18 °C for 1–14 d at week 12.

**Table 3:** Theeffects of broiler breeder dietary vitamin E and egg storage time on the hatchability and embryonic mortality.

Items	Egg Stored 0 d		Egg Stored 14 d ^1^		SEM	*p*-Value		
	Vitamin E 6 mg/kg	Vitamin E 100 mg/kg	Vitamin E 6 mg/kg	Vitamin E 100 mg/kg		Egg Storage	Vitamin E	Interaction
**Week 8**								
Egg weight loss during storage, %	0.33	0.35	2.32	2.36	0.012	<0.001	0.174	0.695
Fertility, %	97.39	94.50	92.44	92.42	0.545	0.004	0.198	0.202
Hatchability of set eggs, %	86.69 ^a^	82.73 ^a^	36.30 ^c^	43.08 ^b^	1.108	<0.001	0.532	0.025
Hatchability of fertile eggs, %	88.95 ^a^	87.48 ^a^	39.20 ^c^	46.66 ^b^	1.009	<0.001	0.153	0.039
Chick weight, g	46.56	46.52	45.98	45.90	0.116	0.017	0.806	0.923
Healthy chick ratio, %	99.10	100.00	96.38	95.65	0.726	0.024	0.956	0.580
Healthy chick number per hen	39.4	38.4	16.1	19.1	0.530	<0.001	0.345	0.066
Embryonic mortality ^2^, %								
Early	4.86 ^c^	6.67 ^c^	46.68 ^a^	40.07 ^b^	0.946	<0.001	0.267	0.039
Mid	0.54	0.83	1.12	1.98	0.275	0.132	0.305	0.616
Late	5.66	5.02	13.00	11.29	0.544	<0.001	0.301	0.629
**Week 10**								
Egg weight loss during storage, %	0.42	0.42	2.24	2.26	0.019	<0.001	0.825	0.758
Fertility, %	96.72	96.45	90.03	89.89	0.821	0.001	0.900	0.969
Hatchability of set eggs, %	88.25	90.44	43.96	53.60	1.047	<0.001	0.011	0.090
Hatchability of fertile eggs, %	91.23 ^a^	93.75 ^a^	48.76 ^c^	59.63 ^b^	0.973	<0.001	0.003	0.044
Chick weight, g	46.04	46.06	45.60	45.32	0.123	0.026	0.604	0.550
Healthy chick ratio, %	98.76	99.39	95.98	97.69	0.507	0.039	0.262	0.601
Healthy chick number per hen	40.0	41.7	19.4	24.3	0.484	<0.001	0.002	0.111
Embryonic mortality ^2^, %								
Early	4.51	3.12	32.32	28.02	1.025	<0.001	0.178	0.522
Mid	0.85	0.58	1.52	0.93	0.315	0.424	0.506	0.812
Late	3.41	2.56	17.40	11.42	0.834	<0.001	0.052	0.142
**Week 12**								
Egg weight loss during storage, %	0.13	0.12	1.42	1.40	0.006	<0.001	0.370	0.520
Fertility, %	95.46	94.23	94.53	91.81	0.640	0.205	0.139	0.566
Hatchability of set eggs, %	87.86	86.64	75.99	79.97	1.068	<0.001	0.526	0.238
Hatchability of fertile eggs, %	92.01 ^a^	91.92 ^a^	80.39 ^c^	86.97 ^b^	0.730	<0.001	0.038	0.033
Chick weight, g	45.69	45.89	44.78	44.33	0.149	<0.001	0.675	0.285
Healthy chick ratio, %	99.31	100.00	99.23	98.84	0.290	0.300	0.800	0.359
Healthy chick number per hen	40.1	40.2	34.6	36.7	0.506	<0.001	0.283	0.351
Embryonic mortality ^2^, %								
Early	6.38 ^b^	5.81 ^b^	16.08 ^a^	9.66 ^b^	0.664	<0.001	0.017	0.042
Mid	0.32	0.99	0.94	1.04	0.306	0.588	0.542	0.646
Late	1.29	1.28	2.59	2.34	0.514	0.268	0.904	0.909

^a^^,b,^^c^ Means with different superscripts in the same row differ significantly (*p* < 0.05). ^1^ The temperature for eggs stored 14 d was controlled at 22–24 °C for 1–14 d at week 8, 22–24 °C for 1–7 d and 16–18 °C for 8–14 d at week 10, and 16–18 °C for 1–14 d at week 12. ^2^ The early, mid, and late embryonic mortality indicated that embryos died between d 1–7 of incubation, d 8–14 of incubation and d 15–21.5 of incubation, respectively.

**Table 4:** The effects of broiler breeder dietary vitamin E and egg storage time on the antioxidant status of egg yolks.

Items ^1^	Egg Stored 0 d		Egg Stored 14 d ^2^		SEM	*p*-Value		
	Vitamin E 6 mg/kg	Vitamin E 100 mg/kg	Vitamin E 6 mg/kg	Vitamin E 100 mg/kg		Egg Storage	Vitamin E	Interaction
**Week 8**								
α-tocopherol (μg/g)	34.25	171.42	34.75	171.08	4.592	0.993	<0.001	0.964
T-SOD (U/g)	384.9	396.6	402.8	357.5	10.750	0.643	0.430	0.208
MDA (nmol/g)	82.37	68.95	73.21	69.68	1.915	0.284	0.039	0.211
T-AOC (μmol/g)	4.67	5.35	3.42	4.12	0.133	<0.001	0.017	0.970
**Week** **10**								
α-tocopherol (μg/g)	39.95	187.83	41.10	191.17	5.060	0.827	<0.001	0.915
T-SOD (U/g)	417.2	448.7	365.2	368.0	8.153	0.001	0.305	0.390
MDA (nmol/g)	71.32	62.32	68.92	60.74	1.497	0.538	0.009	0.864
T-AOC (μmol/g)	4.22	4.85	3.87	4.34	0.097	0.041	0.010	0.673
**Week** **12**								
α-tocopherol (μg/g)	41.15	189.48	41.30	181.90	1.878	0.334	<0.001	0.316
T-SOD (U/g)	395.5	395.1	391.1	381.2	14.912	0.761	0.865	0.876
MDA (nmol/g)	70.72	59.34	72.14	59.99	0.874	0.559	<0.001	0.826
T-AOC (μmol/g)	4.65	5.13	3.64	4.55	0.118	0.003	0.008	0.372

^1^ T-SOD = total superoxide dismutase, MDA = malondialdehyde, T-AOC = total antioxidant capacity. ^2^ The temperature for eggs stored 14 d was controlled at 22–24 °C for 1–14 d at week 8, 22–24 °C for 1–7 d, and 16–18 °C for 8–14 d at week 10, and 16–18 °C for 1–14 d at week 12.

**Table 5:** The effects of broiler breeder dietary vitamin E and egg storage time on the antioxidant status of newly hatched chicks.

Items ^1^	Egg Stored 0 d		Egg Stored 14 d ^2^		SEM	*p*-Value		
	Vitamin E 6 mg/kg	Vitamin E 100 mg/kg	Vitamin E 6 mg/kg	Vitamin E 100 mg/kg		Egg Storage	Vitamin E	Interaction
**Week** **8**								
**Serum**								
T-SOD (U/mL)	240.0	231.6	254.6	236.3	9.447	0.614	0.489	0.795
MDA (nmol/mL)	7.76	7.42	9.00	8.88	0.309	0.041	0.710	0.867
T-AOC (μmol/mL)	1.03 ^b^	1.00 ^b^	1.24 ^b^	1.99 ^a^	0.054	<0.001	0.003	0.002
**Liver**								
T-SOD (U/mgprot)	559.8	568.7	535.4	499.0	12.556	0.075	0.590	0.377
MDA (nmol/mgprot)	0.35	0.36	0.35	0.33	0.019	0.605	0.897	0.730
T-AOC (μmol/10mgprot)	0.96 ^ab^	0.93 ^ab^	0.85 ^b^	1.12 ^a^	0.035	0.577	0.105	0.038
**Yolk sac**								
T-SOD (U/mgprot)	72.7	81.2	74.2	85.3	2.155	0.527	0.036	0.759
MDA (nmol/mgprot)	0.51	0.52	0.57	0.60	0.016	0.042	0.530	0.782
T-AOC (μmol/10mgprot)	0.75	0.97	0.91	1.09	0.029	0.021	0.002	0.692
**Week 1** **0**								
**Serum**								
T-SOD (U/mL)	234.7	266.9	238.7	293.5	10.521	0.476	0.052	0.597
MDA (nmol/mL)	7.61	6.77	8.69	8.39	0.194	0.002	0.156	0.497
T-AOC (μmol/mL)	0.89	1.47	1.41	2.11	0.094	0.006	0.003	0.746
**Liver**								
T-SOD (U/mgprot)	611.3	631.4	556.3	599.5	14.161	0.141	0.278	0.687
MDA (nmol/mgprot)	0.43	0.42	0.40	0.38	0.014	0.288	0.505	0.884
T-AOC (μmol/10mgprot)	0.96	1.10	1.14	1.21	0.035	0.056	0.154	0.626
**Yolk sac**								
T-SOD (U/mgprot)	59.5	72.5	74.0	70.7	2.935	0.295	0.420	0.181
MDA (nmol/mgprot)	0.55	0.48	0.61	0.52	0.016	0.097	0.021	0.762
T-AOC (μmol/10mgprot)	0.74	0.77	0.86	0.87	0.030	0.089	0.720	0.825
**Week 12**								
**Serum**								
T-SOD (U/mL)	246.2	259.6	238.6	267.6	8.678	0.992	0.237	0.657
MDA (nmol/mL)	6.83	6.20	7.48	6.00	0.215	0.600	0.024	0.332
T-AOC (μmol/mL)	0.98	1.43	1.23	1.67	0.048	0.018	<0.001	0.972
**Liver**								
T-SOD (U/mgprot)	586.8	612.1	654.0	648.7	16.746	0.137	0.769	0.653
MDA (nmol/mgprot)	0.50	0.43	0.48	0.42	0.017	0.720	0.058	0.981
T-AOC (μmol/10mgprot)	0.99	0.99	0.88	1.00	0.037	0.464	0.438	0.451
**Yolk sac**								
T-SOD (U/mgprot)	56.1	65.1	57.4	75.3	2.474	0.258	0.013	0.388
MDA (nmol/mgprot)	0.43 ^b^	0.49 ^b^	0.63 ^a^	0.51 ^b^	0.016	0.003	0.290	0.012
T-AOC (μmol/10mgprot)	0.77	1.04	0.79	0.90	0.020	0.142	<0.001	0.062

^a^^,b^ Means with different superscripts in the same row differ significantly (*p* < 0.05). ^1^ T-SOD = total superoxide dismutase, MDA = malondialdehyde, T-AOC = total antioxidant capacity. ^2^ The temperature for eggs stored 14 d was controlled at 22–24 °C for 1–14 d at week 8, 22–24 °C for 1–7 d, and 16–18 °C for 8–14 d at week 10, and 16–18 °C for 1–14 d at week 12.
